# Foliar Application of Sulfur-Containing Compounds—Pros and Cons

**DOI:** 10.3390/plants12223794

**Published:** 2023-11-07

**Authors:** Dimitris L. Bouranis, Styliani N. Chorianopoulou

**Affiliations:** 1 Plant Physiology and Morphology Laboratory, Crop Science Department, Agricultural University of Athens, 11855 Athens, Greece; s.chorianopoulou@aua.gr; 2 PlanTerra Institute for Plant Nutrition and Soil Quality, Agricultural University of Athens, 11855 Athens, Greece

**Keywords:** foliar fertilization, foliar spraying, sulfur-containing adjuvants, sulfur-containing agrochemicals, sulfur-containing metabolites

## Abstract

Sulfate is taken up from the soil solution by the root system; and inside the plant, it is assimilated to hydrogen sulfide, which in turn is converted to cysteine. Sulfate is also taken up by the leaves, when foliage is sprayed with solutions containing sulfate fertilizers. Moreover, several other sulfur (S)-containing compounds are provided through foliar application, including the S metabolites hydrogen sulfide, glutathione, cysteine, methionine, S-methylmethionine, and lipoic acid. However, S compounds that are not metabolites, such as thiourea and lignosulfonates, along with dimethyl sulfoxide and S-containing adjuvants, are provided by foliar application—these are the S-containing agrochemicals. In this review, we elaborate on the fate of these compounds after spraying foliage and on the rationale and the efficiency of such foliar applications. The foliar application of S-compounds in various combinations is an emerging area of agricultural usefulness. In the agricultural practice, the S-containing compounds are not applied alone in spray solutions and the need for proper combinations is of prime importance.

## 1. Introduction

Plants acquire and use carbon dioxide from the atmosphere, as well as water and inorganic nutrients from the soil for their growth, development, and reproduction. Water and nutrients enter the plant body through the root system, and the application of the depleted nutrients in the soil is a common agricultural practice, i.e., soil fertilization. This approach poses limitations when it comes to the accessibility of nutrients for plants. This is primarily due to the formation of insoluble forms in the soil following fertilizer application or leaching of soluble forms through the soil, which can ultimately contaminate ground water sources [1,2].

Leaves have the capacity for water and nutrients uptake when exposed to rain or irrigation. Spraying the above ground part of the plant with dilute solutions of the needed nutrients is also a common agricultural practice, i.e., foliar fertilization. When the supply of a nutrient by the rhizosphere is inadequate or uncertain, the foliar application of fertilizers is widely used in current crop management for optimal production of the crop. Foliar fertilization provides benefits compared to soil fertilization when the demand for nutrients by the plant exceeds the capacity of its root system for nutrient uptake, and/or nutrients within the plant restrict delivery to tissues. It is also advantageous in adverse environmental conditions that may adversely affect crop performance [1]. This practice is used for supplying additional nutrients such as nitrogen (N), phosphorus (P), potassium (K), magnesium (Mg), sulfur (S), and micronutrients. Through foliar fertilization, essential nutrients are supplied to the plant in the proper concentrations, enhancing the plant’s nutritional status and ultimately leading to increased yield quality and production [3,4].

Apart from the nutritional significance of foliar application, there is a second important contribution of this agricultural practice, the biofortification of crop production. The crops are biofortified with elements such as zinc (Zn), iron (Fe), manganese (Mn), selenium (Se), and compounds such as folate and vitamins [5,6,7]. Furthermore, leaves can acquire compounds when they are applied as ingredients of an aqueous solution and foliar application provides a third significant advantage. It is also used to alleviate the detrimental effect of various adverse conditions, and combinations of them including heat, cold, frost, drought, and salinity by spraying the crop with a range of compounds, such as growth regulators and stimulators or biostimulants [8]. These substances play a pivotal role in plant growth and development as well as disease control. In practical terms, foliar application is becoming increasingly vital in agricultural practices.

The nutrients are applied as aqueous solutions via spraying and focusing on the penetration of ionic, polar solutes through the leaf surface. This is a target-oriented fertilization method, and an environmentally friendly one. The nutrients are delivered directly to foliage, and in less amounts compared with soil fertilization. This practice reduces the environmental impact caused by soil fertilization [1,2].

The response of foliage to spraying is in some cases variable and perhaps not reproducible. This can be attributed to the ever-changing environmental conditions during spraying, coupled with a multitude of factors affecting the penetration of the solution sprayed on foliage. This complex scenario has been aptly referred to as the “spray and pray” situation by Fernández and Eichert (2009) [9]. The environmental conditions, including the temperature, relative humidity, and wind speed, influence the first step of foliar application. Other factors could be the molecular weight, along with the physical and chemical properties of the nutrient, the concentrations of the active ingredients, the time of application, and the environmental conditions. These factors affect the penetration of the spray solution through the plant surfaces, and in particular the penetration through the structures of cuticle and stomata [9,10,11].

Another factor that affects the penetration of the spraying material is the morphological and anatomical construction of the leaf. The leaf surface physiology, and especially the presence of the epicuticular waxes, determine the rate of retention, the wettability and finally the penetration of the spraying material [12,13,14]. The effectiveness of the application is assessed in relation to its penetration, the reduction in the deficiency or its correction, the improvement in yield, and the quality of the produce [15,16,17]. The foliar application of nutrients is an agricultural tool that supplements soil fertilization in conditions of lower availability of these nutrients in the soil, and especially at the critical times of nutrient demand [15].

The commercial solutions for foliar application of nutrients are generally composed of at least two major components, the active ingredient(s) and the adjuvant(s). Adjuvants are materials that help the spraying solution and its ingredients to improve the wetting, spreading, and sticking on the surface of foliage, and then to support the rate of the penetration of the applied nutrients. The challenges associated with the penetration of the applied mineral nutrients during foliar have prompted the extensive utilization of adjuvants and ongoing research to discover new ones that can improve the effectiveness of spray treatments. The addition of the adjuvant modifies the physical and chemical properties of the spray solution towards an effective wetting of the leaf surface [18].

Sulfur nutrition plays a crucial role in the growth and development of higher plants and the S demand of agricultural crops may be from 1 kg S t^−1^ for sugar beet up to 17 kg S t^−1^ for Brassica crops [19]. S supply has consequences for crop productivity and nutritional quality and S-fertilization has become an issue. The problem is the reduced industrial emissions of S to the atmosphere, which results in the decreased deposition of S onto agricultural land in many areas of the world [20]. Sulfur limitation results in decreased yields and quality parameters of crops [21]. Adequate S nutrition is also required for plant health and resistance to pathogens [22]. A series of specific responses aimed at optimizing acquisition and utilization are induced by sulfur limitation in all plant species studied to date [21,23,24]. Factors affecting S supply and the subsequent impacts on crops have been discussed by Haneklaus et al. (2007) [19]. On the other hand, complex interactions occur between S and other nutrients at the level of the whole plant; the individual tissues and the cellular compartments have been discussed by Courbet et al. (2019) [25]. S deficiency mainly acts by reducing plant growth, which in turn restricts the root uptake of N, K, and Mg and vice versa. In legumes, the nodules show high requirements for S and there is a strong interaction between N and S.

It is a common phenomenon for the crops to develop under a harsh agricultural environment in the region of cultivation. Climate change, in combination with other factors, built a cultivation environment that causes reductions in crop yields due to various adverse conditions. The foliar application of natural compounds improves the obtained yield under the circumstances, including plant metabolites [8]. Applying a range of metabolites in crops with valuing properties and functions, such as glutathione, proline, glycine betaine, citric acid, L-tryptophan, polyols, ascorbic acid, lipoic acid, and tocopherol, contributes to the crop’s ability to tolerate various abiotic stresses it encounters at different developmental stages. The benefits of such applications are assessed and evaluated by measuring or determining their effects on morphological parameters, along with biochemical, metabolic, and genetic ones, in a variety of crop plants. Such applications usually result in improved yields when applied in the field. Foliar application of plant metabolites has proven to be an effective way to support the tolerance of the crop to the various abiotic stress [8]. Within the various tested plant metabolites so far, several S-containing compounds seem to be of prominent importance and have been incorporated in the foliar fertilization practice.

**Figure 1:**
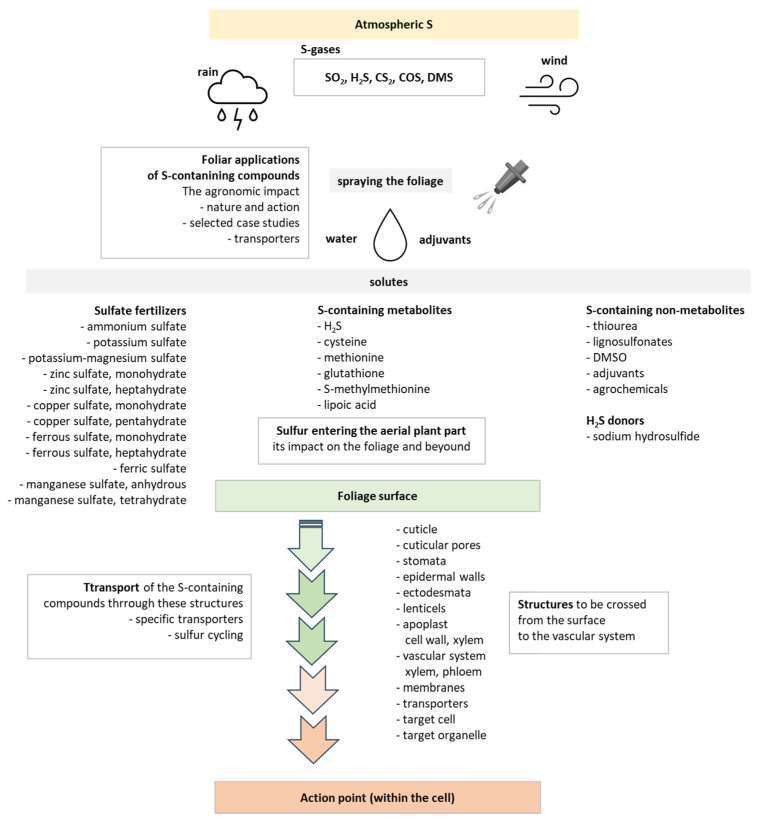
Foliar application of S-containing compounds. These compounds are S-gases [sulfur dioxide (SO_2_), hydrogen sulfide (H_2_S), carbon disulfide (CS_2_), carbonyl sulfide (COS), dimethyl sulfide (DMS)], fertilizers containing sulfate, S-containing metabolites, and S-containing non-metabolites. For the integrity of the approach, in the last group, the S-containing adjuvants and agrochemicals have been added. The arrows indicate the complex journey of the S-containing solute to the action point within the cell which includes several structures to cross, and the characteristics of each one of them is summarized in the text. Towards understanding and handling the effectiveness of such foliar applications, the nature and mode of action of these compounds, along with some characteristic case studies, are discussed.

The target of this review is to deepen in the foliar application of the S-containing compounds. The significance of the foliar application of such compounds on the crop for nutritional purposes, along with the biofortification of crop production and the mitigation of the various negative results of the stresses, have provided significant research progress. Several S-containing compounds have been used as biostimulants to support the crop to overcome stressful conditions. These compounds are the metabolites cysteine (Cys), methionine (Met), glutathione (GSH), S-methylmethionine (SMM), and lipoic acid (LA), as well as the non-metabolites thiourea (TU) and lignosulfonates, sodium sulfite, and sodium hydrogen sulfide. The latter two produce hydrogen sulfide. For each of these compounds, we discuss on their role and contribution to crop growth, development, and health after foliar application (Figure 1).

## 2. The Journey of the Sprayed Compounds from the Aerial Plant Surfaces to the Action Point within the Plant Tissues and Cells

In plants, the root is the organ that absorbs water and inorganic nutrients from soil, which are then transported to the leaf tissues and cells through the vascular system. In leaves, the inorganic nutrients are incorporated or assimilated into various organic compounds and the leaves act as sources that send these organic compounds to the other plant organs, which in turn act as sinks. The acquisition of inorganic nutrients by the roots is in mutual relationship with the assimilation activities in leaves. The plant foliage which comprises leaves, stems, inflorescences, flowers, and fruits can take up nutrients as well [26].

### 2.1. From the Surface to the Vascular System

The journey of a compound sprayed on the aerial plant surfaces up to the action point within the cells starts with its penetration and transport through the following structures: the various layers of the cuticle, the cuticular pores, the stomata, the epidermal walls and the ectodesmata, as well as the lenticels; and continues through the cell wall, and the apoplast, to cross the various membranes, to enter cells, then to enter the phloem and circulate within the vascular system for long-distance transport, and again to cross membranes, to enter cells and finally to reach the action point [1,5,9,26]. In this section, we summarize the contribution of these structures to the journey of the sprayed compound, and then we focus on the penetration and transport of the S-containing compounds that are sprayed on a surface of foliage and enter the corresponding epidermis.

A leaf consists of petiole and blade with an upper and a lower surface. Both surfaces are covered with epidermis, the outside material of which is the cuticle, a composite material. This is the first barrier for the penetration of the spraying solution, i.e., water and the solutes that carries, through the leaf surface. The cuticle consists of various layers; it is composed of lipids which are embedded into the matrix of the biopolymer of cutin. Hydrocarbons, along with fatty acids, primary alcohols, and esters, have been identified as major constituents of cuticle. The cuticular layers are protective layers, mainly composed of cutin, waxes, polysaccharides, and phenolics, with various combinations. The existence of these layers makes the leaf surface waterproof. The changes in the quantity and chemistry of the cuticular waxes during the developmental stages of the leaves influence the wettability and the permeability of the leaf cuticles [27,28,29,30].

As ingredient of the spray, sulfate is an anion, and the outer layer of the cuticle is negatively charged due to carbonyl and carboxyl groups existed within the cutin. This layer presents the major resistance to penetration, whilst in the inner layer the mobility of ions is greater than in the outer one. In contrast, the penetration of non-charged, lipophilic solutes through the cutin occurs by dissolution and diffusion. The mechanism of the penetration of polar, hydrophilic molecules has been discussed by Fernández and Eichert (2009) [9]. Two parallel pathways in cuticles seem to be responsible for the transport of the lipophilic substances and the hydrophilic ones, with separate diffusion paths for the lipophilic non-electrolytes and the hydrated ionic compounds. The presence of cracks on the cuticular surface, i.e., the cuticular pores, contributes to the penetration of the solutes. Inside the pores, the cations are attracted to the negative charge and diffuse passively. In this way, the electrical charge is progressively balanced. In parallel, the anions start to penetrate through the pores. The rate of diffusion of ions across the layers depends on their concentration gradient. The cuticle is a polyelectrolyte presenting isoelectric points at approximately 3.0. The ion-exchange capacity of the cuticle alters the fluctuations of the pH Spraying with solutions with pH values higher than 3.0 renders the cuticle negatively charged. On the surface, this corroborates the diffusion of the cations, whilst it repels the anions, such as sulfate. Alshaal and El-Ramady (2017) [5] have provided an estimation of the time for 50% needed for the entry of nutrients into the plant leaf tissue, according to which sulfate needs 8 days to enter the leaf tissue. For comparison, Mg^2+^ needs 2–5 h, K^+^ 10–24 h, Ca^2+^, Zn^2+^, and Mn^2+^ 1–2 days, whilst Fe^2+^ or Fe^3+^ 10–20 days to enter the leaf tissue. Phosphate anion needs 5–10 days, and molybdate 10–20 days.

In the ledges of the cuticle, aqueous pores are found preferentially at the basal cells of trichomes, guard cells, and in anticlinal walls. Such pores are dynamic in nature and are formed only in the presence of water because hydration of the permanent dipoles and the functional ionic groups is needed. The distribution of polar groups within the lipophilic polymer leads to the formation of either isolated aqueous clusters or a continuous aqueous space where the polar groups are spread out. The radii of these pores range from 0.45 to 1.18 nm. If the diameter of the molecule of the sprayed solute is within this range or less, it is likely that this compound can penetrate through the aqueous pores and reach the epidermal cell walls. The polar characteristics of the plant surface results in more efficient translocation of cations rather than anions. An increase in cation valency, decreases the rate of cation penetration through the cuticle. A charge-neutral molecule like urea, can pass easily and efficiently through the plant surface [26,31].

Once the nutrient penetrates the plant surface, the barriers to nutrient uptake after the cuticle layers are cell walls and plasma membranes; then the nutrient can take either the apoplastic or the symplastic pathway to reach the vascular tissues for its translocation. The cell wall includes primary and secondary one, along with middle lamella. The primary cell wall is composed of pectin along with polysaccharides, the cellulose, and hemicelluloses. This results in a negative charge on the surface due to carboxyl and hydroxyl groups. There are structures in the epidermal walls that terminate on the surface of the outer epidermal cell walls, called ectodesmata. These structures are usually present on both the upper and lower epidermal cells, the guard cells, the sides of the larger leaf veins, trichomes and epidermal cells surrounding the capitate hairs. Ectodesmata are always covered by the cuticle and do not extend to the outer leaf surface. In this region, the structure is comparatively loose compared with the structure of the cell wall, whilst the interspaces are filled with coarse reticulum of cellulose, extended from the plasmalemma to the cuticle. In this way, ectodesmata serve as a polar pathway in absorption and excretion of substances. In cuticles, most of these pore-like structures have a diameter of less than 1 nm and distributed at a density approximately 1000 pores/cm^2^. These allow ready accessibility to low-molecular-weight solutes but not to larger molecules like synthetic chelates [26,32,33,34].

Stomata are found in the surface of the leaves, i.e., dynamic openings composed by guard and subsidiary cells that facilitate the gaseous exchange of water vapor, CO_2_ and O_2_ between the external atmosphere and the internal one in the stomatal cave. Each of the upper and the lower surface of a leaf, the adaxial and the abaxial, respectively, consists of numerous stomata. The stomatal density is usually higher on the lower surface, whilst fewer or no stomata may be present on the upper surface. The solutes penetrate through stomata by diffusion along the wall pores, which are less selective and thus offer less resistance as compared to the cuticle. The overall contribution of stomata to the foliar uptake process seems to serve as a major passage to ion penetration. A higher density of cuticular pores in cell walls between the guard and subsidiary cells leads to a higher absorption of nutrients [26,35].

The nutrients diffuse across the cell wall against a concentration gradient and reach the apoplast. Apart from the cell walls, the apoplast also includes the intercellular space and the xylem, which consists of dead cells. The nutrients are transported into the xylem sap and allocated to the leaves via the flow of water. Several functions take place in the apoplast: water and nutrient transport, cellulose synthesis, the transport of materials for the construction of the cuticle, and molecules that are involved in plant development and in plant responses to various adverse conditions. Compounds secreted by pathogens can also be found in the apoplast. The diversity of the molecules found in the apoplast highlights its importance in the survival of plant cells [27]. The characteristics of the apoplast are expected to influence the fate of nutrients applied to foliage by spraying. The pH value ranges from 4.5 to 7.0 depending on plant species and growing conditions. The H^+^-ATPases and the ABC-transporters located at the plasma membrane pump H^+^ into the apoplast, thus lowering its pH. The apoplast has a role in ionic balance and serves as a transient ion reservoir. The functions of the leaf apoplast include the regulation of transient pH fluctuations, signal transduction leading to stomatal responses, and detoxification of toxic elements. The ion relation in the apoplast varies temporally because of changes in the metabolic activity caused by the day and night transition. Immediately after onset of light, the process of photosynthesis leads to an alkalization of the apoplastic pH. The apoplastic pH can be affected by external factors such as drought or flooding. It is therefore expected that the rate of diffusion of ions into the apoplast would be determined by the pH [26,36,37,38,39,40,41,42,43,44,45].

Χylem and phloem constitute the vascular system of the plant, which contributes to long-distance transport. Main function of the vascular system is the effective distribution of nutrients between plant organs. The phloem connects sources with the sinks of the photosynthetic products. Within the phloem sap, inorganic cations and anions are co-transported. In annual plants, usually the mature leaves are sources for carbon and nutrients that are supplied to sinks, including roots, flowers, and seeds. In perennial plants, the source-sink relationship depends on the season. During spring, buds and developing leaves are sinks for carbon and nutrients. Later in the year, mature deciduous leaves are the source. Stem tissues of bark and wood can be source organs in spring and sink organs during active growth and leaf senescence [46,47,48,49,50].

Also, the importance of the vascular system as a structure that is useful for inter-organ communication [51] that contributes to the transfer of signals from roots to shoot, and vice versa, has been highlighted—for example, by signaling nutrient demand. Signaling from and to other plant organs such as flowers, seeds, bark, and wood in the case of perennial plants is considered too. The compounds that are transported through the vascular system create cycling pools among the various tissues and organs. These pools utilize the transport processes over the plasma membrane of cells. They are also influenced by the short-distance transport processes through the organellar membranes within cells, which in turn results in intracellular cycling, including that of S [39,50,52,53,54,55,56,57,58,59].

### 2.2. The Fate of the Foliar-Applied S-Containing Compounds

Sulfate and the S-containing compounds Cys, GSH, Met, γ-glutamylcysteine (γEC), and SMM have been detected as being transported within the vascular system. All these compounds can cycle within the plant tissues, between roots and the shoot and vice versa, and can be distributed from places of surplus to places of demand. In the xylem sap, S mainly exists as sulfate. Reduced S-containing compounds have also been found but in lower amounts. S-containing compounds play a critical role in the response of plants to abiotic stress factors. For example, although abscisic acid (ABA) is the key regulator of responses to drought and/or high-salt stress, it seems that there is an interaction of S-metabolism and ABA biosynthesis. It has been reported [60] that sulfate supply affects synthesis and steady-state levels of ABA in Arabidopsis, and evidence has been provided for a significant co-regulation of S-metabolism and ABA biosynthesis that operates to ensure sufficient Cys involved in the acting mechanism, a fact that highlights the importance of S for stress tolerance of plants. In addition, it has been tested [61] whether sulfate is an early xylem-delivered signal communicating drought stress to the shoot. Sulfate and ABA concentration changes in the xylem sap were related to stomatal conductance. It was found that drought affects sulfate transporter expression. Xylem-delivered apoplastic sulfate induces stomatal closure, and triggers gene expression in guard cells in an ABA-like manner. The xylem-derived sulfate seems to be a chemical signal of drought that induces stomatal closure via anion channels, and/or guard cell ABA synthesis.

The composition and concentrations of the S compounds differ with S nutrition, during the season, during the developmental stage, along the trunk, between species, and due to mycorrhization. These aspects have been discussed for trees in detail [48,50,62,63,64,65,66]. The supply of reduced S from shoot to roots is carried out by the phloem transport. Together with S allocation in the xylem, the cycling pools of the circulating S compounds provide oxidized and reduced S to respective sinks [48,50,67]. Thus, the above-mentioned S-containing compounds can cycle between the interconnected structures: cells, tissues, and organs. Cycling occurs among various tissues and includes exchanges between phloem and xylem, both within the shoot and in transit to stems and roots. This process facilitates the distribution of S to the sites of its demand for growth and development, helps signal the S status of the plant, and plays a crucial role in regulating the overall nutrition of the entire plant. Sulfur cycling has been investigated in perennial plants regarding the annual growth cycle. Perennial plants need to store nutrients during dormancy, and they must mobilize nutrients during spring to supply the newly sprouting shoot with carbohydrates, N, S compounds and needed nutrients [39]. As an example of S cycling at the tissue level, cycling of Cys and GSH has been shown in maize plants between mesophyll and bundle sheath cells. Sulfate reduction and assimilation up to Cys occurs in the bundle sheath cells, whilst the use of Cys in GSH synthesis takes place in the mesophyll cells [48,50,68].

To reach their site of action, the S-containing compounds need to cross membranes too. Therefore, specific transporters are needed at the whole plant level. Sulfate uptake is a dynamic biological process that occurs at the cell, tissue, and organ levels. Sulfate transporters (SULTR) are integral membrane proteins controlling the flux of sulfate entering the cells and the subcellular compartments across the membranes. Takahashi (2019) [69] has elaborated on sulfate transport systems. Sulfate is necessary for the synthesis of many metabolites, and Gigolashvilli and Kopriva (2014) [70] have discussed the various transporters that contribute to S metabolism. The primary and secondary assimilation, the biosynthesis, the storage, and the final utilization of S-containing compounds all require movement between organs, tissues, cells, and organelles. Therefore, efficient transport systems of S-containing compounds are required across the plasma membrane and the organellar membranes. A detailed understanding of the mechanisms and regulation of transport is needed towards improving the plant yield, biotic interactions, and nutritional condition of crops, along with the successful biofortification of the involved pathways. A brief presentation of the corresponding transporters that are known takes place in the discussion of the foliar application of each S-containing compound.

## 3. The Impact of the Atmospheric S on Foliage

Atmospheric sulfur comprises a range of gaseous forms of S including sulfur dioxide (SO_2_), hydrogen sulfide (H_2_S), carbon disulfide (CS_2_), carbonyl sulfide (COS), dimethyl sulfide (DMS) [71] and aerosols, mainly as sulfate from the oxidation of S gases or organosulfates [72,73] that enter the plant. It has long been recorded that several nutrients including S and S-compounds are absorbed by the leaves following rain and transported to other parts. S-compounds are released into the atmosphere by the emission of industrial gases or during the ore-smelting activity [34]. When SO_2_ is emitted into the atmosphere and transported by wind and air currents, it reacts with water, oxygen, and other chemicals to form sulfuric acid, which in turn mixes with water and other materials and falls to foliage. Winds can blow SO_2_ over long distances, thus making sulfuric acid-containing rain a global problem. Although a portion of the SO_2_ in acid rain is from volcanoes and other natural sources, most of it comes from the burning of fossil fuels. When SO_2_ is in high concentrations in rain and acidic fog, this negatively affects foliage. In the short term, the plants are less able to absorb sunlight because they become weak and less able to withstand freezing temperatures. In the long term, foliage contains dead leaves. Moreover, acid rain leaches aluminum, which may be harmful to plants and crops as well as animals, and nutrients from the soil [74]. Global climate change increases the occurrence of wildfires worldwide and fire-smoke has a devastating effect on the entire ecosystem including plants over very long distances. The concentrations of released SO_2_ into the atmosphere during fires can reach 3000 nL L^−1^ [75].

SO_2_ is a highly hazardous gas, posing severe stress on plants due to sulfite formation in the apoplastic space, which in turn leads to necroses of the leaves. It enters the leaves mostly via the stomata and is converted to sulfite, a toxic substance, and the elevated sulfite must be detoxified. Studies investigating the detoxification mechanism(s) of SO_2_ in natural environments and non-model organisms are plentiful. Baillie et al. (2016) [76] studied the detoxification of the S surplus in plants and discussed different strategies of survival. It was found that plants from selected fields reacted differently after exposure to SO_2_ at the level of (i) the closure of the stomata and (ii) the overall accumulation of thiols and/or sulfate. In a strategy, channeling of the S surplus took place by the formation of S-metabolites like thiols, i.e., the strategy of reductive detoxification. Oxidative detoxification into sulfate was also found in another strategy, with or without increased sulfite oxidase activity. Baillie et al. (2019) [77] provided new insights into sulfite detoxification, as they found apoplastic peroxidases that enable additional sulfite detoxification, acting as a first line of defense upon exposure to SO_2_. Sulfite detoxification is of utmost importance in plants, and it has been found that the sulfite concentration is strictly controlled. Increased guaiacol peroxidase activity can act as a first line of defense and assists the activity of sulfite oxidase. Weber et al. (2021) [75] examined the detoxification of S surplus in the leaves from beech (*Fagus sylvatica*) and oak (*Quercus robur*) due to exposure to elevated SO_2_. In both species, an induced stress reaction was indicated by a 1.5-fold increase in oxidized GSH. In beech leaves, the activities of the sulfite detoxification enzymes were increased by 5 fold and a trend of sulfate accumulation was observed. In contrast, oaks did not regulate sulfite oxidase and apoplastic peroxidases during smoke exposure; however, the constitutive activity was 10-fold and 3-fold higher than in beech. Beeches use the efficient upregulation of oxidative sulfite detoxification enzymes, while oaks hold a constitutively high enzyme pool available.

Plants emit gaseous S compounds. Bloem et al. (2012) [78] focused on the dependence of H_2_S and COS exchange with ambient air on the S status of oilseed rape (*Brassica napus* L.) as well as on the fungal infection of oilseed rape with *Sclerotinia sclerotiorum*. S fertilization and fungal infections affect the exchange of H_2_S and COS from the crop. Such emissions are related to the plant S status or the fungal infection. H_2_S was either released or taken up by the plant depending on the ambient air concentration and the plant demand for S. The emissions of H_2_S were closely related to both the pathogen infections and S nutrition. The S fertilization caused a shift from H_2_S consumption by S-deficient oilseed rape plants to H_2_S release after the addition of S. The fungal infection caused a stronger increase in H_2_S emissions. COS is normally taken up by plants. Healthy oilseed rape plants acted as a sink for COS, whilst fungal infection caused a shift from COS uptake to COS releases. Jing et al. (2019) [79] have elaborated on the exchange of carbonyl sulfide (COS) and carbon disulfide (CS_2_) between the atmosphere and cotton fields in an arid area. The vegetation presented a predominant seasonal net uptake of COS during the growing season. This was not the case for the CS_2_, where its fluxes from the vegetation presented no significant seasonal variations. The exchange rates of CS_2_ and COS were found to be stimulated by the addition of urea fertilizer.

## 4. S-Containing Mineral Compounds Applied as Foliar Sprays

Inorganic S-containing compounds may contain sulfate as the anion, or the thiol group -SH. There is a good number of sulfate compounds that have been used as fertilizers for foliar application, namely ammonium sulfate, potassium sulfate, potassium-magnesium sulfate, zinc sulfate (monohydrate or heptahydrate), copper sulfate (monohydrate of pentahydrate), ferrous sulfate (monohydrate or heptahydrate), ferric sulfate tetrahydrate, and manganese sulfate (anhydrous or tetrahydrate). Sodium hydrosulfide (NaHS) is used as donor to produce hydrogen sulfide.

### 4.1. Spraying with Sulfate Salts

The importance of applying sulfate by foliar application. To correct nutrient deficiencies by foliar fertilization, the soluble sources of the various nutrients are more efficient compared to insoluble or slightly soluble sources, and sulfate salts are soluble. The principal sources of macro- and micronutrient fertilizers and their solubility, including the sulfate-containing ones, have been presented and discussed by Fageria et al. (2009) [2]. The chelated sources of micronutrients are comparatively more efficient compared to non-chelated ones, but between them the chelated sources are expensive. The selection of appropriate sources of inorganic fertilizers for foliar sprays is of great importance, considering penetration and transport efficiency, and foliage burning. Considerable differences have been reported among the fertilizer sources, as regards burning of foliage following the foliar application of inorganic fertilizers, especially N. The risk of foliage burning is more likely when the N source is other than urea, such as ammonium sulfate [80]. Leaf injury and yield depression of soybean by the various NPKS materials were noted when the fertilizer application was taking place during midday rather than the early morning or late afternoon hours [81]. This piece of information clearly shows that the evaporation rate of the sprayed drops onto foliage condenses the drop, thus altering the pH; therefore, application timing is of importance. Foliar application should be made when the plant is not under water stress, i.e., when the plant is turgid and cool [82]. When the crop is under a given nutrient stress, this is the most critical time to apply it. Stress periods occur during active growth activity, especially when the plant is switching from the vegetative to the reproductive stage [2].

Sulfate salts are used for agronomic biofortification, through foliar application of the micronutrient fertilizer directly to the leaves. Rice, wheat, maize, legumes, sorghum, millet, and sweet potato dominate diets worldwide and agronomic biofortification is mainly focused on them [83,84]. Foliar fertilization with micronutrients often stimulates more nutrient uptake and efficient allocation in the edible plant parts than soil fertilization [85]. Foliar pathways are generally more effective in ensuring uptake into the plant because immobilization in the soil is avoided. However, the combination of soil and foliar application is in several cases the most effective method [86,87]. The downside of foliar application is that fertilizers can easily be washed off if rain follows, whilst several fertilizers are more costly and difficult to apply [88].

Zinc-enriched grains are of great importance for crop productivity on potentially Zn-deficient soils, as they secure better seedling vigor, denser stands, and higher stress tolerance. Due to its high solubility and low cost, zinc sulfate (ZnSO_4_) is the most widely applied inorganic source of Zn. Foliar, along with combined soil and foliar application of Zn fertilizers under field conditions, are highly effective and very practical ways and uptake and accumulation of Zn at the level of whole wheat grain is maximized. Such applications can achieve concentrations up to 60 mg Zn kg^−1^ [89]. The most effective method for increasing Zn in grain was the combination of soil plus foliar application method that provided a 3.5-fold increase in the Zn concentration of grain. The timing of foliar Zn application is an important factor determining the effectiveness of the foliar-applied Zn fertilizers in increasing grain Zn concentration. According to Cakmak (2008) [89], large increases in loading of Zn into grain can be achieved when foliar Zn fertilizers are applied to plants at a late growth stage.

The main inorganic source of Fe is FeSO_4_, while Fe_2_(SO_4_)_3_ is also used. In a case study provided by Papadakis et al. (2007) [90], three-months-old citrus plants, including two genotypes [sour orange (*Citrus aurantium* L.) and Carrizo citrange (*C. sinensis* L. cv. Washington navel × *Poncirus trifoliata*)], were sprayed with 0.018 M iron sulfate (FeSO_4_·7H_2_O), or 0.018 M manganese sulfate (MnSO_4_·H_2_O). After its foliar application, Mn was found to be relatively mobile within citrus plants, resulting in a significant increase in Mn concentrations in top leaves, basal leaves, stems, and roots of sour orange, and in top leaves, basal leaves, and stems of Carrizo citrange. Transport of Mn from the basal, sprayed leaves to the top, unsprayed ones were found for both genotypes. The results did not provide any evidence for Mn translocation from sprayed tissues to roots. As regards Fe, it was found to be strictly immobile within citrus plants after its foliar application. Spraying with Fe significantly increased the concentrations of Fe in the stems and basal leaves of both genotypes and no transport of Fe from sprayed tissues to unsprayed ones (top leaves, and roots) was found [90].

Iron chlorosis is a very common nutritional disorder in plants and variable results have been reported from Fe source studies related to differences in Fe placement, rate, time of application, weather, and soil conditions [91,92,93]. The most effective agronomic practices for the Fe enrichment of crops are through foliar application of mineral Fe. Foliar application has already showed to increase Fe concentrations in wheat grain and rice grain [94]. However, there are also contradictory results, as some studies have showed no response of plants upon foliar Fe application, especially under treatment with inorganic and chelated Fe fertilizers [87,88].

Fe and Cd present similar chemical properties and entry route and are closely correlated in crops cultivated in contaminated soils. Many studies have characterized the effects of Fe in crops under Cd stress, and Afzal et al. (2021) [95] highlighted the fact that the underlying mechanisms that reduce the Cd concentration within the plant when different Fe fertilizers are applied are poorly understood. A report by Bashir et al. (2018) [96] suggested that foliar application of Fe complexed with lysine significantly increased plant growth and biomass, biochemical and physiological attributes in *O. Sativa* grown in Cr stress environment. Wang et al. (2021) [97] supplied foliar Fe fertilizers in the ionic (FeSO_4_·7H_2_O, Fe(NO_3_)_3_·7H_2_O), and chelated forms, (Na_2_FeEDTA, and FeEDDHA) and it was concluded that foliar application of chelated ferrous Fe provides a promising alternative approach for enhancing growth and controlling Cd accumulation in rice plants. These results indicate that foliar application of chelated ferrous Fe provides a promising alternative approach for enhancing growth and controlling Cd accumulation in rice plants. The above-mentioned references contribute to the understanding of the associations between plant Fe nutrition status and Cd accumulation.

Sulfate transport and transporters. The amount of sulfate ions that enter the cell to be metabolized in the cytosol, chloroplast, or plastid, or to be stored in the vacuole, depends on the expression levels and the functionalities of the existing sulfate transporters (SULTR, see Section 2.2) in the corresponding membranes. The entire system for sulfate transport requires different types of SULTR. When sulfate penetrates the leaf epidermis, the corresponding the tissue and cell type transporters are expressed. The regulation takes place at the transcriptional and post-transcriptional levels and controls the expression levels of the corresponding SULTR, towards optimal internal distribution in response to the availability of sulfate and the demand for the synthesis of various S metabolites [69]. Several SULTR have been detected in the vasculature and the xylem parenchyma of leaf [98,99]. The occurrence of SULTR types varies depending on the location at the organ, cell, or subcellular compartment levels, the environment, and the genotype [100]. There has been research towards answering whether these transporters release sulfate into the xylem, as well as on the transporters that are involved in the efflux of S compounds into the xylem from xylem parenchyma cells and from storage tissues along the trunk [48]. Sulfate contents, together with SULTR expression, have been investigated in leaves, bark, and wood of field-grown poplar, and control of sulfate cycling by SULTR expression has been revealed [61,101]. Sulfate accumulates in bark and wood during autumn, whilst sulfate can be taken out of the xylem or phloem sap for storage [50].

### 4.2. The Foliar Application of Hydrogen Sulfide

The impact of atmospheric H_2_S. The H_2_S applied to foliage can be used as a S source for growth. Being lipophilic in nature, H_2_S can rapidly cross the membranes without the intervention of channels. When no sulfate is supplied to the root, plants can grow with atmospheric H_2_S as the sole S source. Within the plant, H_2_S is biologically reactive, directly incorporated into Cys. The H_2_S applied to foliage can be potentially phytotoxic. Intensive research through the years showed that the various plant species differ considerably in the susceptibility of the applied H_2_S. Prolonged exposure to 0.03 L H_2_S L^−1^ air inhibited the biomass production of sensitive dicot species. This concentration is a realistic one for industrially and agriculturally polluted areas. Monocot species can tolerate up to 1.5 L H_2_S L^−1^ air without negative effects on plant biomass production. A probable explanation of the tolerance of these species to elevated H_2_S is that the meristem is sheltered by leaves and H_2_S can hardly penetrate the meristem [73,102,103,104,105,106].

Fumigation of plants with H_2_S. The fumigation of plants with H_2_S has proven to be a powerful way to obtain insights into the regulation of sulfate uptake and assimilation. Upon H_2_S fumigation, shoot Cys and GSH contents increase significantly, indicating that absorbed H_2_S is metabolized with high affinity in these thiols. Sulfate-deprived plants may fully alleviate the development of S-deficiency symptoms by receiving foliar H_2_S. Many plant species have been tested and the rate of the foliar H_2_S uptake followed Michaelis–Menten kinetics. Kinetics is controlled by the rate of incorporation of H_2_S into Cys [107,108].

The regulation of sulfate metabolism in barley (*Hordeum vulgare*) seedlings exposed to atmospheric H_2_S in the presence and absence of a sulfate supply has been studied by Ausma and De Kok (2020) [106]. Sulfate deprivation resulted in reduced shoot and root biomass production, decreased shoot and root total S contents, decreased sulfate content, and lower Cys, GSH, and soluble protein levels. On the other hand, it resulted in increased shoot and root molybdenum content, increased APS reductase (APR) activity and increased expression and activity of the root sulfate uptake transporters, and enhanced dry matter, nitrate and free amino acid contents [106]. Barley could use the absorbed H_2_S by foliage as a S source for growth. In fact, barley switched S source, from rhizospheric sulfate to atmospheric H_2_S. The development of S-deficiency symptoms was alleviated in sulfate-deprived barley exposed to 0.6 μL L^−1^ atmospheric H_2_S. Fumigation of both sulfate-deprived and sulfate-sufficient plants with H_2_S downregulated APR activity, as well as the expression and activity of the sulfate uptake transporters. The sulfate utilization in barley seems to be controlled by signals originating in the shoot [106].

Donors of H_2_S. Donors of H_2_S were studied in recent years for their contribution as oxidative stress reducers. Such molecules seem to contribute to cellular signaling, and are post-translational modifiers. The same holds true for several derivative compounds of H_2_S such as polysulfides and polysulfanes. The H_2_S donor compounds are explored in the agricultural field towards possible applications in improving the productivity and quality of crops [73]. To date, exogenous applications have been carried out using chemicals capable of delivering H_2_S; the standard chemicals are sodium hydrosulfide (NaHS) and inorganic sodium polysulfides (Na_2_S_x_) such as Na_2_S_2_, Na_2_S_3_, and Na_2_S_4_ [108]. In aqueous solutions, the delivery of H_2_S by the above-mentioned polysulfides depends on the pH and the corresponding pKa. NaHS is a short-lived donor that does not mimic the slow continuous process of H_2_S generation in vivo. NaHS is the cheaper solution and has been sprayed directly on plants in a wide range of concentration [109]. The foliar application of H_2_S donors to different plant species at different stages of development can alleviate damage caused by abiotic stress. Physiological features including post-harvest preservation of vegetables are enhanced [109,110,111,112]. The positive results that have observed so far suggest that further basic research on the foliar application of H_2_S donors is reasonable and highly required. So far, it has been found that H_2_S mediates in signaling and in the increase in tolerance to different stresses including water deficit, salinity, high temperature, and increased concentrations of heavy elements (Cd, Cr, Cu, Al, As) [73,113,114,115,116,117,118,119,120]. H_2_S in higher plants may be part of a mechanism of response to environmental stress conditions [108]. H_2_S and reactive sulfur species (RSS) interact with reactive oxygen (ROS) and reactive nitrogen (RNS) species, and relevant signaling molecules [111,121]. The set of the reactive chemical species seems to form a cellular network of redox signals [122].

A possible signaling mechanism where H_2_S contributes is the formation of persulfides or hydrosulfides (RSSH) from the protein cysteine residues, as several enzymes, transcription factors, and channels seem to be involved in the mechanism(s) [123,124]. H_2_S auto-oxidises in the presence of O_2_ and SO_3_^2−^, S_2_O_3_^2−^, SO_4_^2−^ and polysulfanes are formed [70]. H_2_S is precursor of biological polysulfides [125]. Polysulfanes, polysulfides (with S_n_ > 2), and RSSH present a diversity of oxidation states between the S atoms, a trait that allows the molecules to present a dual character as oxidants and reducers. This diversity probably contributes to a multifunctionality character of the signaling of H_2_S and the derived compounds. H_2_S presents reactivity comparable to that of GSH against H_2_O_2_ and free radicals. However, its value as a cellular antioxidant is limited because low concentration of H_2_S in vivo is found [73,126,127]. The signaling properties of the endogenously generated H_2_S within the plant cells are mainly observed during persulfidation, a protein post-translational modification (PTM) that affects the redox-sensitive cysteine residues [108].

## 5. S-Containing Metabolites Applied to Foliage by Spraying

Several S-containing metabolites have been used as biostimulants, towards supporting the crop to overcome various stressful conditions. The metabolites Cys, Met, GSH), SMM, and LA are discussed in this section. For each of these compounds, we summarize their role(s) and contribution to crop growth, development, and health after foliar application. A summary of the case studies on the foliar applications of S-containing metabolites discussed in this section is given in Table 1.

### 5.1. Spraying with L-Cysteine

The nature and action of L-cysteine. Cys is a reductive amino acid with a thiol side chain, incorporated into proteins as residue with structural function. In parallel, it is precursor for biomolecules, such as GSH, vitamins, and defense compounds (glucosinolates and thionins) [123,124,128]. The various oxidative conditions that put plants under stress are characterized by the production of various reactive species: oxygen, nitrogen, and sulfur ones (ROS, RNS, and RSS, respectively). Many of these reactive species either damage different types of macromolecules or serve as messengers. Due to the reactivity of the thiol group, some protein Cys residues are prone to oxidation by these molecules. The modification of Cys thiol groups contributes either to catalytic and regulatory functions, or to protective, redox signaling mechanisms. Reversible redox post-translational modifications (PTMs) of physiological relevance are disulfide bonds, S-nitroso thiols, sulfenic acids, S-glutathione adducts, thiosulfinates, S-persulfides and S-sulfenyl-amides. Coturier et al. (2013) [129] have reviewed the variety and the physiological roles of these PTMs, which are mostly controlled by two oxidoreductase families, the thioredoxins and the glutaredoxins.

Case studies of foliar application of cysteine. Cys is a natural compound that has been used as a component in foliar applications. One aspect of application is towards alleviating the adverse effect of salinity stress on different plant crops. Foliar application of Cys can ameliorate negative effects of salt stress on plants, and the following two examples are provided.

Perveen et al. (2018) [130] assessed the effect of Cys, on maize crop (*Zea mays* L., var. Malka and hybrid DTC) under salt stress, among various S-containing compounds. Maize plants were subjected to salt stress treatment (0 vs. 90 mM NaCl). Two weeks after the salt stress application, plants were sprayed with different levels of the following compounds, i.e., Cys (20 mM), FeSO_4_ (10 mM), LiSO_4_ (10 mM), and a mixture at a 1:1:2 ratio, against control (non-spray). Foliar application was performed twice at one-week interval after the salt stress treatment. The salinity stress significantly decreased the growth of both maize genotypes. The foliar application of Cys decreased the relative water content (in var. Malka) and the contents of free proline, glycine betaine, and flavonoid. Among the used compounds, a differential response was observed in increasing the growth parameters of both maize genotypes, where FeSO_4_, LiSO_4_ and the mixture with a 1:1:2 ratio, were better than the foliar application of Cys alone [130].

In alleviating the effect of salinity stress on soybean crop, Sadak et al. (2020) [128] investigated the contribution of Cys (20 mg L^−1^ and 40 mg L^−1^) by carrying out experiments during two successive summer seasons at 30 and 45 days from sowing, in soybean plants grew under salinity conditions (3000 mg L^−1^, and 6000 mg L^−1^). Salinity caused decreases in soybean growth parameters, the photosynthetic pigments, the N, P, K contents, along with the yield and yield components, and percentage of oil. Cys treatments improved the growth and yield of soybean plant either irrigated with tap water or saline water and presented a beneficial role in alleviating the adverse effect of salinity stress on soybean plant. It was suggested that the spraying solution of 40 mg L^−1^ of Cys is the most effective treatment [128].

Another line of work on the topic focused on the agronomic biofortification of broccoli, which serves as a functional food because it can accumulate Se, glucosinolates, the well-known bioactive amino-acid-derived secondary metabolites, and polyphenols. The chemical and physical properties of Se are very similar to those of S, and competition between sulfate and selenate for uptake and assimilation has been demonstrated. Towards an efficient agronomic biofortification of broccoli florets, Bouranis et al. 2023 [131] studied the working question whether it is possible to overcome this competition by exogenously applying Cys along with Se application. Broccoli plants were cultivated in a greenhouse; and at the beginning of floret growth, the application of Se (0.2 mM) was coupled with the application of Cys (0.05 mM). Foliar application of this combination and isodecyl alcohol ethoxylate (IAE) as adjuvant provided 298 μg Se per floret (which is an acceptable Se concentration); dry mass, organic S concentration and carotenoids were increased. When silicon ethoxylate (SiE) was used as adjuvant the outcome was different; Se concentration was 313 μg Se per floret and Sorg was decreased, whilst carotenoids, total chlorophylls, and glucosinolates were increased.

Cysteine transporters. Although plants possess many amino acid transporters, many of them capable of transporting Cys, some even with a high specificity [132,133], it is not clear whether Cys transport is less specific through general amino acid permeases. In general, amino acid transporters act on the specificity of substrates, but there is much information about the S-containing amino acid Cys transporters. For instance, Arabidopsis UMAMIT14 is a broad substrate transporter for amino acids, whereas not a specific transporter for Cys [134]. In plants, protein synthesis occurs in three organelles and the intracellular transport of amino acids is essential [70]. However, this may not be the case for Cys, the synthesis of which is also localized in cytosol, mitochondria, and plastids [135], although it has been found that the synthesis of Cys can be restricted to a single compartment without affecting survival and with only small effects on growth [70,136]. Cys also undergoes intercellular transport, although its contribution to a total long-distance flow of S may not be very high [137]. Seeds can assimilate sulfate and therefore they do not depend on transport of Cys [138]. In C4 plants, S nutrition is dependent on intercellular Cys transport, since sulfate is reduced in the bundle sheath cells only and Cys is the transport metabolite from these cells to mesophyll and other cell types of the leaves [139]. Thus, the molecular nature of Cys transport into the cells as well as in mitochondria and plastid membranes remains obscured.

### 5.2. Spraying with Glutathione

The nature and action of glutathione. GSH (γ-L glutamyl-L-cystinyl-glycine) is natural, bioactive compound present in most plant tissues and involved in diverse aspects of plant metabolism. It is a tripeptide consisting of glutamic acid (Glu)—cysteine (Cys)—glycine (Gly). GSH is a powerful antioxidant, involved first and foremost in the removal of ROS. ROS increase when plants cope with different abiotic adverse conditions, and the increasing oxidative damage to nucleic acids, proteins, and lipids will affect various metabolic activities. The degree of damage depends on the balance among the production and removal of ROS through the scavenging mechanisms. Towards mitigating the oxidative damages, a complex defensive antioxidant system is in action, which includes antioxidant enzymes such as SOD, POX, APX, GR and non-enzymatic antioxidants such as GSH, ascorbate, and phenolic compounds. Some plants exhibit a variation in GSH, called homoglutathione, with the same biological properties [8,140,141,142].

GSH is an important pool of reduced S, a central component of the glutathione—ascorbate cycle, and a predominant non-protein thiol present in plant cells. It contributes to the regulation of many cell functions, including the synthesis and repair of DNA, the synthesis of proteins, activation, and the regulation of enzymes in plants. GSH is the precursor of phytochelatins (PCs), which are GSH-containing oligomers able to chelate heavy metals, thus contributing to the sequestration of heavy metal and transport to the vacuole. PCs are involved in flower development and plant defense signaling [143,144,145,146,147]. Thus, it is clear that GSH is crucial for biotic and abiotic stress management [146].

Case studies of foliar application of GSH. Various treatments with GSH have been performed as foliar applications, with target to increase plant tolerance to different abiotic adverse conditions. According to Akram et al. (2017) [148], when GSH was applied to the leaves of various varieties of salt-stressed soybean (*Glycine max*) plants, significant increments in plant growth and production parameters were observed in comparison to plants under salt-stressed conditions only as control. Compared with control, the number of seeds per plant increased depending on the genotype examined, whilst pods per plant and yield per plant, parameters that impact on crop yield, were significantly improved. Genotypes categorized as susceptible to salt stress presented better responses when also treated with GSH [148].

According to Nakamura et al. (2019), foliar application of GSH to oilseed rape plants (*Brassica napus*) cultured hydroponically, affected the distribution and behavior of Zn [147]. The treatment significantly increased the Zn content in shoots, the root-to-shoot Zn translocation ratio, the Zn concentration in the cytosol of root cells, and enhanced xylem loading with Zn. Following the foliar GSH treatment, the gene encoding pectin methylesterase was upregulated in roots and signals triggered in response to foliar-applied GSH increased Zn availability in roots and mobilized Zn from the root cell wall. Root-to-shoot translocation of Zn was activated and increased Zn accumulation in the shoot was found. These findings suggest that the foliar application of the reduced form of GSH improved the Zn mobilization and transport within the plant [147].

According to Ghoname et al. [146], foliar application with 50 or 100 mg L^−1^ of GSH vs. arginine, or tryptophan on hot pepper (*Capsicum annuum* L. cv. Albasso) resulted in significant increases in plant growth parameters and yield, an increase in the level of IAA, GA3, decrease in ABA content, accompanying by increases in ascorbic acid, anthocyanins, tannins, phenolic compounds, carbohydrate content, protein content and amino acids composition, all parameters of nutritive value. The authors concluded that, comparatively, the promoting effect of GSH and arginine treatments, especially at 100 mg L^−1^, was more pronounced than that of tryptophan treatments [146].

The effect of the combined application of GSH and ascorbic acid, on growth, yield, and yield components of two cultivars (Sakha93 and Giza168) of wheat plant (*Triticum aestivum* L.) was studied by El-Awadi et al. (2014) [149]. In this approach, the antioxidants were foliar applied twice at the two concentrations of 50 and 100 ppm. The first spray was applied 30 days after sowing and the second one at 15 days later (45 days after sowing). Applying both antioxidants at 100 ppm improved wheat growth and yield, accompanied by increase in yield and yield components of the two cultivars, along with increases in photosynthetic pigments, carbohydrate, total free amino acids, and protein contents [149].

Sadak et al. (2017) [150] evaluated the role of foliar treatment with GSH in enhancing the antioxidant defense system of chickpea plant under different levels of seawater salinity. Increasing concentrations (50, 100 and 150 mg L^−1^) of GSH were tested. Foliar application of GSH caused significant increases in the contents of osmo-protectants of chickpea plants. Compared with the plants irrigated with tap water, the examined levels of diluted seawater significantly increased H_2_O_2_ and lipid peroxidation. The different concentrations of GSH resulted in significant decreases in H_2_O_2_ contents and lipid peroxidation levels in the control and salinity-stressed plants. It was concluded that foliar application of GSH was effective in improving chickpea performance in several aspects by reducing H_2_O_2_ free radical, enhancing compatible osmolytes and antioxidant enzyme activities, and providing membrane stability. Marked increases in the activities of the antioxidant enzymes ascorbate peroxidase, glutathione reductase, peroxidase, and superoxide dismutase were observed in plants treated with GSH at 100 mg L^−1^ either under normal irrigation or salinity-stressed conditions [150].

Rehman et al. (2021) [151] investigated the potential of GSH at the level of 1 mM in combination with moringa leaf extract (MLE; 3%) in wheat under salt stress. GSH served as an antioxidant and the extract as an organic biostimulant. The combination applied in sequence as seed priming and foliar application on wheat growth. The sequential application of MLE and GSH improved osmotic stress tolerance. The positive results were due to stabilized membrane integrity, decreased electrolyte leakage, and enhanced endogenous GSH and ascorbate levels [151].

Jung et al. (2019) [152] investigated the effects of exogenously applied GSH to the leaves of *B. napus* seedlings exposed to 10 μM Cd. Foliar GSH treatments took place at the concentrations of 50 (162.7 μM) or 100 mg kg^−1^ (325.4 μM) of. In this case study, 2 mL L^−1^ of a commercial surfactant (20% sodium lignosulfonate and 10% polyoxyethylene alkyl aryl ether) was incorporated in the foliar solution. The foliar application of GSH to Cd-stressed *B. napus* seedlings reduced Cd-induced ROS levels by increasing seedling AsA, GSH, and NADPH concentrations, thus enhancing the antioxidant-scavenging defenses and the redox regulation. The results demonstrated that GSH improved plant redox status by upregulating the AsA-GSH-NADPH cycle and reestablishing normal hormonal balance. Therefore, GSH can potentially be applied to Cd-polluted soil for phytoremediation purposes [153].

Glutathione transporters. GSH is present in all compartments, and its concentration is high, especially in the mitochondria [154]. Moreover, GSH is subject to long-distance transport [126]. The presence of GSH transporters in the plasma membrane has long been recognized, although the molecular nature of these carriers is still an open field for research. Some transporters of the oligopeptide transporter family can transport GSH. However, for the high flux of GSH within plant cells, their affinity and specificity were not as high as expected [155,156,157]. As an alternative pathway for GSH transport, it has been proposed that the components of GSH are moved across the membrane, through the combination of its degradation, amino acid transport, and synthesis in the new compartment [158]. The key enzyme in this scenario is gamma-glutamyl transferase (GGT). It is present in plants, and it is important for the recovery of apoplastic GSH [159]. GGT is localized on the apoplast side of plasma membrane or in the tonoplast, and therefore it cannot be responsible for the intracellular GSH transport.

### 5.3. Spraying with Methionine

The nature and action of L-methionine. Met is an S-containing amino acid, which contributes to a diversity of physiological functions. Met is an essential amino acid in humans, and it could be safely added to food, except infant foods [160]. It regulates transpiration, the photosynthetic rate, and protein synthesis; it maintains membrane stability and relative water content; it reduces ROS production, H_2_O_2_, and MDA contents; it enhances enzyme activities that protect plant cells from oxidative damage under water-deficit conditions [161,162]. Thus, it is an effective regulator of plant growth and development under water deficit [163].

Case studies of foliar application of Met. Met has been used as a component in foliar applications towards alleviating the adverse effects of drought stress and salinity stress, and the following examples are presented.

The effects of Met, among other amino acids, were studied by El-Bauome et al. (2022) [160] on cauliflower plants (cv. Arasya) grown under well-irrigated and drought-stressed conditions. After transplantation, all plants were acclimated for a month by keeping them at 60–70% field capacity. The control group was sprayed with distilled water plus 0.05% (*v*/*v*) Tween-20 as a wetting agent, whilst the Met group was sprayed with Met (25 mg L^−1^) plus 0.05% (*v*/*v*) Tween-20. Foliar treatments were performed 5 times at 30, 45, 60, 75, and 90 days after transplanting; then the pots were left to grow for additional 15 days. Compared with the untreated plants, foliar application of Met significantly increased height, diameter, freshness, dry matter, leaf area, leaf chlorophyll content, leaf relative water content, vitamin C, proline, total soluble sugar, reducing sugar, and nonreducing sugar. On the other hand, polyphenol oxidase (PPO), peroxidase (POD), and phenylalanine ammonia-lyase (PAL) were significantly reduced. A similar trend was observed in glucosinolates, abscisic acid (ABA), malondialdehyde (MDA), and total phenols [160].

In another example provided by Maqsood et al. (2022) [164], two wheat genotypes were grown with 100% field capacity (FC), the control treatment, up to the three-leaf stage. The 25-day-old seedlings of two wheat genotypes (Galaxy-13 and Johar-16) were subjected to 40% FC, the water-deficit stress treatment, with and without foliar application of 4 mM Met. The foliar application of Met substantially improved growth, photosynthetic, and gas exchange attributes under water-deficit conditions in both genotypes. Under the stress conditions, the Met application improved K, Ca^2+^, and P contents, whilst the activities of SOD, POD, and CAT were further enhanced [164].

The impact of foliar-applied Met on growth and performance of okra was investigated by Zulqadar et al. (2015) [165]. Different levels of Met were applied to okra (5, 10 and 20 mg L^−1^), 15 and 30 days after sowing. For foliar application, 0.1% Tween-20 was used as a wetting agent. It was concluded that foliar application of Met could be effective in inducing more flowering and promoting the growth and yield of okra. Foliar application of Met at the level of 10 mg L^−1^ had a significant effect on the growth, yield and physiological parameters of okra as compared to untreated control. It increased the number of flowers, number of fruits, root length, shoots fresh and dry weight, the photosynthetic rate, chlorophyll contents and fruit yield up to 77, 96, 71, 64, 65, 71, 60 and 64%, respectively [165].

The responses of four maize genotypes (FH1275, FH 936, FH 1231, and FH 1227) to the foliar application of two Met levels (5 and 10 mg L^−1^) under 80 mM NaCl stress were studied by Shahid et al. (2021) [166]. Salinity was applied at four leaves stage and maintained at 80 mM gradually. After 7 days of salinity application, spray with Met was applied with four days difference. Yield parameters were examined at plant maturity, and the Met level of 10 mg L^−1^ showed better results as compared to 5 mg L^−1^, both under saline and non-saline environments [166]. The use of Met seems to be a cost-effective treatment.

Such foliar applications may include other (natural) compounds too as in the following examples. Almas et al. (2021) [167] studied physiological and yield attributes of tomato by foliar spray of Met, and/or L-phenylalanine (Phe) under saline stress (4, and 6 dS m^−1^). The tomato plants were sprayed with Met (0.01% and 0.02%), Phe (0.01% and 0.02%), and their combination at the vegetative growth stage. The foliar application of Met induced salt resistance and improved all the growth and yield parameters. The combination of Met plus Phe displayed higher carotenoid contents, total carbohydrates, total free amino acids, and proline contents compared with the only salt-treated plants. The combined application of both amino acids reduced the lipid peroxidation rate and electrolyte leakage [167].

Towards an efficient agronomic biofortification of broccoli florets, Bouranis et al. (2023) [131] (see Section 5.1 for Cys) studied the working question whether it is possible overcome the competition between sulfate and selenate by exogenously applying Met along with Se application. Broccoli plants were cultivated in a greenhouse and at the beginning of floret growth the application of Se (0.2 mM) was coupled with the application of Met (0.1 mM). Foliar application of this combination and IAE as adjuvant provided 156 μg Se per floret (which is an acceptable Se concentration); organic S concentration and carotenoids were increased. When SiE was used as adjuvant the outcome was different; Se concentration was 156 μg Se per floret and Sorg was decreased, whilst carotenoids, total chlorophylls, and glucosinolates were increased. The combinations of Met (0.1 mM) with Cys (0.05 mM), or with phenylalanine (0.25 mM) and tryptophane (0.05 mM) along with Se (0.2 mM), were also studied [131], as Met, phenylalanine and tryptophane are all precursors for the biosynthesis of glucosinolates. Again, the nature of the adjuvant differentiated the responses. When IAE was used, FM was decreased (a negative result from the commercial point of view), whilst Se was found within acceptable contents. Car and total chlorophylls were increased but the total glucosinolates. In the case of SiE as adjuvant, the organic S decreased, fresh mass was not affected, whilst Car, total chlorophylls and total glucosinolates increased.

Methionine transporters. Very little is known about methionine transporters. A complex demand for inter- and intracellular transfer is needed for Met, particularly when its derivatives are considered [70], and Met must be transported to all compartments with protein synthesis. Cytosolic Met synthase is involved in the regeneration of Met in the SAM cycle [61,159]. As mentioned, the amino acid transporters act on the specificity of substrates, and the UMAMIT14 of Arabidopsis is a broad substrate transporter for amino acids, whereas not specifically for the transport of Met, as in the case of Cys [134].

### 5.4. Spraying with Alpha Lipoic Acid

The nature and action of lipoic acid. LA (6,8-dithiooctanoic acid) is a S-containing compound with powerful oxidizing properties in both of its forms, the reduced dihydrolipoic acid (DHLA) and the oxidized one (LA) [168,169,170,171]. It is a coenzyme of several key enzymes involved in the regulation of the redox status of plants, and the energy metabolism in eukaryotes [170,171]. It plays a significant role as a cofactor for pyruvate dehydrogenase and glycine decarboxylase, components of certain mitochondrial enzyme complexes [172]. LA application provided salinity tolerance by stimulating antioxidant enzyme activities in plants [173,174], whilst under abiotic stresses, including salt stress, the oxidized and reduced forms of LA decreased in shoots of wheat and barley [175,176].

Case studies of foliar application of LA. LA has been used as component in foliar applications towards alleviating the adverse effects of various stresses on different crop plants. Yildiz et al. (2015) [174] investigated the effects of LA on NaCl toxicity, proteomic, biochemical, and physiological changes in the leaves of canola (*Brassica napus* L.) seedlings. The applied concentration of LA alleviated the toxic effects of salinity stress by decreasing MDA content and increasing growth parameters, cysteine content, and activities of CAT and POD. Out of 28 proteins that were differentially expressed, 21 proteins were successfully identified that significantly upregulated or downregulated. These proteins were related to photosynthesis, energy metabolism, protein folding and stabilization, signal transduction, and stress defense. The authors concluded that foliar application with LA is an effective application for improving growth of canola under salinity stress [174]. Elkelish et al. (2021) [177] studied a combination of LA plus Cys in wheat, i.e., the influence of LA in a grain dipping pre-cultivation treatment, in combination with Cys as a foliar application, under well-watered or deficit irrigation. The authors concluded that applied LA at 0.02 mM as seed soaking treatment, combined with Cys at 50 ppm as a foliar application could be considered as a successful application in wheat cultivation under water-deficit conditions, by providing physiological tolerance and restoring yield attributes in wheat [177].

Transporters of LA. As regards the transport of LA and corresponding transporters, to our knowledge, there is no such information available.

### 5.5. Spraying with S-Methyl Methionine

The nature and action of S-methylmethionine. SMM is a non-proteinogenic amino acid synthesized from Met and S-adenosylmethionine (SAM or AdoMet), and the reaction is catalyzed by Met S-methyltransferase (MMT). SMM participates in methylation processes within the cell and plays an important role in the transportation and storage of sulfur [61]. It serves as methyl donor for Met synthesis from homocysteine, catalyzed by the homocysteine S-methyltransferase (HMT). MMT and HMT together have been proposed to constitute an SMM cycle that protects the free Met pool from depletion by an overshoot in SAM synthesis. During the Met cycle, SMM can revert to Met through a transmethylation reaction involving homocysteine [178]. The SMM cycle operates throughout the plant [179]. SMM is produced by all angiosperms and is involved in their S metabolism [180,181]. It contributes to the regulation of the levels of both Met and SAM. Plants lack the negative feedback loops that regulate SAM pool size in other eukaryotes. The SMM cycle may be the main mechanism whereby plants achieve short-term control of the SAM level [179].

Apart from having an important role in the S metabolism, SMM is involved directly or indirectly in the stress and disease tolerance of plants. The role of SMM in the biosynthesis of sulfopropionates (that serve as osmoprotectants) and polyamines is valuable for plant resistance [180]. It can moderate the damaging effects of various stressors by enhancing the production of dimethyl sulfopropionate, which acts as an osmo- and cryo-protectant [182], by increasing the biosynthesis of polyamines, and by regulating ethylene production [178,183,184]. SMM is highly effective in protecting against cold stress, it stimulates the phenylpropanoid pathway, it increases the content of phenol derivatives and anthocyanins, and protects the photosynthetic apparatus [185,186].

Case studies of foliar application of SMM. Trials with foliar application of SMM are rare. As an example, provided by Fodorpataki et al. (2021) [187], the effect of foliar application of SMM has been investigated in canola (*Brassica napus* L. cv. Cindi) plants exposed to moderate or severe salt stress for different periods. Canola is a moderately salt tolerant plant, but high salinity inhibits germination of seeds, vegetative growth of young plantlets, and reduces biomass production. After two weeks of development, plants were sprayed in leaves and stem with an aqueous solution of 1 mM SMM. The applied level of SMM alleviated the reduction in the net photosynthetic rate, enhanced the water use efficiency and contributed to the reduction in oxidative membrane damage in fully developed young leaves [188].

SMM transporters. The importance of specific transporters for SMM cycling has been highlighted [50]. The consequences of enhanced phloem loading capacity of SMM has been described by Tan et al. (2010) [65]. Enhanced phloem loading of SMM was mediated by the yeast MMP1 gene that produces an SMM transporter when it was targeted to the phloem and the seeds in pea plants. Over-expression of this gene increased SMM content in phloem exudates; however, SMM did not accumulate in roots. Instead, the expressions of SULTR and APR increased. The downregulation of APR and other genes of the sulfate reduction pathway in leaves corresponded to higher SMM contents. This might function as a signal to reduce sulfate assimilation. Shoot biomass of transgenic pea plants increased, along with the soluble and total seed N content. This study shows that manipulation of long-distance transport can influence whole plant physiology. It was suggested that enhanced xylem loading could be responsible for no accumulation of SMM in roots of these mutants [65].

A summary of the case studies on the foliar applications of S-containing metabolites discussed in this section is given in Table 1.

**Table 1:** Summary of the case studies on the foliar applications of S-containing metabolites discussed in Section 5. Cys: cysteine, GSH: glutathione, Met: methionine, LA: lipoic acid, SMM: S-methylmethionine, IAE: isodecyl alcohol ethoxylate, SiE: silicon ethoxylate, Phe: phenylalanine, and Tyr: tyrosine.

Crop	Agricultural Situation	Compound	mM	mg L^−1^ (or %)	Foliar Application	Adjuvant	Reference
maize	salinity stress	Cys	20		alone or		[130]
					combined with		
					FeSO_4_ & LiSO_4_		
soybean	salinity stress	Cys	0.17	20	alone		[128]
			0.33	40			
broccoli	Se biofortification	Cys	0.05		combined	IAE	[131]
					with Se	SiE	
soybean	salinity stress	GSH	2		alone	Tween-20	[148]
oilseed rape	Zn biofortification	GSH	100		alone	Triton-X	[147]
hot pepper		GSH	0.16	50	alone		[146]
			0.33	100			
wheat		GSH	0.16	50	combined		[149]
			0.33	100	with AsA		
chikpea	salinity stress	GSH	0.16	50	alone		[150]
			0.33	100			
			0.49	150			
wheat	salinity stress	GSH	1		combined with		[151]
					moringa leaf extract		
Brassica napus	Cd-polluted soil	GSH	0.16	50			[152]
	phytoremediation	GSH	0.33	100			
cauliflower	drough stress	Met	0.17	25	alone	Tween-20	[160]
wheat	drough stress	Met	4		alone		[164]
okra		Met	0.03	5	alone	Tween-20	[165]
			0.07	10			
			0.13	20			
maize	salinity stress	Met	0.03	5	alone		[166]
			0.07	10			
tomato	salinity stress	Met	0.67	0.01%	alone or combined		[167]
			1.34	0.02%	with Phe		
broccoli	Se biofortification	Met	0.1		combined with	IAE	[131]
			0.1		Cys, or Phe + Tyr	SiE	
canola	salinity stress	LA			alone		[174]
wheat	drough stress	LA	0.02		combined		[177]
					with Cys		
canola	salinity stress	SMM	1		alone		[187]

## 6. S-Containing Non-Metabolites Applied as Foliar Sprays

In this section, we discuss the foliar application of thiourea, the lignosulfonates, the usefulness of dimethyl sulfoxide, and the S-containing adjuvants. For the reader to receive an integrative picture, the S-containing agrochemicals that have been used so far are mentioned.

### 6.1. Spraying with Thiourea

The nature and the action of thiourea. The plant growth regulators (PGRs) are chemical compounds that modulate the responses of plants under biotic and abiotic stresses at the cellular, tissue, and/or organ levels. Thiourea (or thiocarbamide; TU) is a synthetic PGR-containing nitrogen as -NH2 (36%,) and sulfur as -SH (42%). TU has three functional groups, the amino, imino, and thiol ones, each with biological roles. It is characterized by high-water solubility and quick absorption in living tissues and has gained wide attention for its role in plant stress tolerance, where it seems that it modulates several of the involved mechanisms [188,189]. The application of TU modulates various physiological responses and mechanisms during development. It is involved in leaf gas exchange, plant water relations, photosynthesis, nutrient assimilation, and enhances the source-to-sink relationship resulting in increased crop yield. TU acts as a thiol-based scavenger of ROS. At the biochemical level, it is involved in antioxidant defense systems, nitrogen and proline metabolism, improves the metabolism of sugars, and protein biosynthesis. At the molecular level and regardless of the applied stress, the application of TU modulates the pattern of gene expression. It upregulates the expression of genes involved in encoding antioxidant enzymes, ROS-activated ion channels, and the regulation of redox state. Also, genes involved in calcium signaling, aquaporins and osmotic adjustment, metabolite biosynthesis, and hormonal regulation. Moreover, it is involved in the post-transcriptional regulation to enhance the expression of defense-related genes by the synchronization of microRNAs and hormones. Signaling of gene expression is a likely mechanism induced by TU, especially in ABA and calcium signaling events [26,190,191,192,193,194,195,196,197,198,199]. Therefore, TU has been increasingly used to improve plant growth and productivity under normal and stressful conditions.

Case studies of thiourea in foliar applications. Foliar application of TU seems to be more effective under environmental stress than under normal conditions and is more effective in the tissues where it is applied, in improving plant growth and development under heat stress, drought, salinity, and heavy metal toxicity, to a differential extent [190].

Wheat under drought stress. The application of TU resulted in a significant improvement in the growth and photosynthetic efficiency of wheat crop, by increasing vegetative growth, protein content, and yield in wheat under drought stress. TU seems to be an efficient osmo-protectant towards shielding the plants from different abiotic stresses, including drought and heat stress. Tolerance induced by TU is attributed to greater nutrient uptake coupled with the production of osmolytes, improved metabolic processes, and antioxidant defense mechanisms [200,201,202,203].

Heat tolerance in canola. The improvement of heat tolerance in canola with the foliar application of S has been studied by Waraich et al. (2022) [204] with TU as a S source. The design of the experiment included two varieties of canola (Hyola-401 and 45S42), two levels of foliar TU treatments (0 ppm vs. 500 ppm, and two temperature levels (18 C vs. 28 C). Heat stress was imposed at the stage of anthesis, and TU was applied at the same stage. The photosynthetic rate, stomatal conductance and intercellular CO_2_ concentration were improved in TU treatments under the situation, while transpiration rate was decreased with the foliar application of TU. Yield and yield components increased with the foliar application of TU at the level of 500 ppm. Among the genotypes, Hyola 401 performed better following the foliar application of TU under heat stress conditions [204].

Nutritional-quality-related traits of bread wheat. The effect of foliar-applied TU on the growth, yield, and nutritional-quality-related traits of bread wheat has been investigated by Sher et al. (2021) [205] on sandy loam soils in semiarid regions. The treatments of TU levels (500 mg L^−1^, and 1000 mg L^−1^) were applied on two diverse wheat cultivars (Gandam-1 and Galaxy-2013) at tillering, booting, and heading. TU treatments significantly affected the growth, nutritional quality, and morphological traits, and the interaction of the two factors was significant. The application of TU improved the productivity and nutritional quality in both cultivars. Galaxy-2013 performed best at 1000 mg L^−1^ TU application at the heading stage for both productivity and nutritional-quality related traits.

Late sowing of wheat. The potential of TU for enhancing the performance of late sown wheat has been studied by Zain et al. (2017) [202]. Wheat (cv. Galaxy-2013) was sown in mid-December, and two foliar treatments of TU (300 and 600 mg L^−1^) were applied at tillering, jointing, and booting, under water spray and no spray as double control. The foliar application of TU at the tillering stage and level of 300 mg L^−1^ significantly enhanced wheat growth, the number of productive tillers, number of grains per spike, 1000- grain weight and grain yield. The foliar application of TU reduced the harmful effects on late sown wheat [202].

TU and boron toxicity. The relation of TU and nitric oxide (NO) in mitigating the boron toxicity (BT) has been assessed by Kaya et al. (2019) [199] in bread wheat (*Triticum aestivum* L. cv. Pandas) and durum wheat (*Triticum durum* cv. Altıntoprak 98) plants. Plants were grown under 0.05 mM B (control) and 0.2 mM B (BT treatment) supplied to nutrient solution for 4 weeks after germination. Then, foliar application of TU at the concentrations of 200 mg L^−1^ or 400 mg L^−1^ was applied once a week during the period of stress. TU, on the one hand, improved the plant growth, led to a further increase in NO in the leaves, and enhanced enzyme activities but, on the other hand, reduced the contents of soluble sugars, soluble protein, and phenols [199].

### 6.2. Spraying with Lignosulfonates

The nature and action of lignosulfonates. Lignosulfonates (LS) are by-products of the pulp and paper industry, generated by breaking the lignin network during the sulfite pulping process of wood. LS are randomly branched polyelectrolytes, and the water-solubility of them is ensured by the abundance of sulfonate and carboxylic acid groups, the content of which is variable. The properties of LS can be tailored by controlling the production parameters, fractionation, and subsequent modification. Agriculture is among the fields of application of this technical lignin material. The use of LS in agriculture and strategies for the implementation of LS in soil have been discussed by Wurzer et al. (2022) [206,207]. As regards the foliar application of LS, the following are documenting the usefulness of LS in this strategy.

Case studies for foliar application of LS: (i) Iron-lignosulfonates (Fe-LS). The environmental concerns regarding the use of synthetic chelates to overcome iron chlorosis have increased and enforced the search for new and environmentally friendly ligands, including the LS. The LS complexes are less costly per unit of micronutrient but usually less effective than the synthetic chelates. However, the efficacy of the LS products is variable [91,93,208,209]. The formation of a complex between Fe and LS involves different coordination sites. The target here is the efficiency with which the complex will provide iron under various agronomic conditions. Fe coordination environment and speciation have been studied in various LS complexes, in relation to the Fe-complexing capacities, and the chemical characteristics of the different products. According to Carrasco et al. (2012) [93], when Fe(ΙΙ) is used to prepare the Fe-LS product, the complexes form weak adducts, and are sensitive to oxidation at neutral or alkaline pH. In contrast, both Fe(ΙΙ) and Fe(ΙΙΙ) are found when Fe(ΙΙΙ) is used to form the complexes. Reductive sugars are normally present in LS and the content of these sugars favors a higher content of Fe(ΙΙ), even in the case where these complexes prepared using Fe(ΙΙΙ). It seems that the strong Fe(ΙΙΙ)-LS complexes are preferred for application to the leaf [93].

Rodriguez-Lucena et al. (2009) [92] tested the ability of Fe-LS complexes to support plants with Fe through foliar application. Spraying with Fe(III)-LS vs. Fe(III)-EDTA to Fe-deficient cucumber plants showed that uptake and reduction rates of Fe between these complexes were similar. In the case of Fe-deficient tomato leaves, when Fe(III)-LS was used, a similar reduction rate compared with Fe(III)-EDTA was observed, along with a lower uptake rate. Therefore, foliar-applied Fe-LS can be used as an alternative to synthetic chelates, as a valid, cheap, and eco-compatible in dealing with Fe chlorosis. Focusing on the physico-chemical characteristics and the efficacy of different LS, Rodriquez-Lucena et al. (2011) [209] compared eucalyptus LS against spruce LS, a hardwood LS against a softwood one, respectively. All tested LS presented a good ability to complex Fe, whilst the spruce LS was the only one capable to maintain significant amounts of soluble Fe above pH 8. The efficacy of foliar-applied Fe-LS in chlorotic cucumber (*Cucumis sativus* L. cv Ashley) plants was tested in comparison with FeSO_4_ and Fe(III)-EDTA. The Fe content of plants sprayed with Fe-LS was very low compared with the EDTA treatment, but not the biomass and the rates of re-greening. Modifications in the eucalyptus LS improved the efficacy for Fe chlorosis recovery to levels like those found for the spruce LS. The two applications of the LS were recommended [209].

(ii) Foliar application of Zn-lignosulfonates. Zn uptake and localization at the leaf cellular level have been studied by Minnoci et al. (2018) [210], in green bean plants (*Phaseolus vulgaris* L., cv. Linera) after foliar application of a Zn-lignosulfonate (Zn-LS) complex on the oldest leaves at 6 h, 4 days and 30 days, in comparison with a Zn-EDTA chelate. Significant differences in Zn penetration inside the leaves were observed. The Zn-LS complex showed the fastest absorption after 6 h, along with significant differences in Zn localization inside the leaf tissues, and the mesophyll presented the highest absorption of Zn-LS. Zn was detected at the highest concentration in the mesophyll of leaves treated with Zn-LS also at the day 4 and day 30, whereas in those treated with Zn-EDTA it was in the lower epidermis. The treatment with Zn-LS caused an increase in the total thickness of the leaf and of the spongy mesophyll [210].

### 6.3. The Contribution of Dimethyl Sulfoxide as Additive in Spraying Solutions

The nature and action of DMSO. Dimethyl sulfoxide (DMSO) seems to be of specific interest for agronomical use, discussed by Kumar et al. (1976) [211]. DMSO is an excellent solvent, and it can easily solvate the cations because of the negatively charged oxygen atom in its molecule. It easily breaks the hydrophobic non-covalent bonds in the membranes and increases cell permeability. This property contributes to the increase in ion penetration into plant tissues. DMSO forms hydrogen bonds and it can affect (enhance or suppress) enzymatic activities [212,213]. The following case studies highlight the effects of DMSO in various foliar applications.

Case studies of foliar application of DMSO: (i) DMSO and Zn. Kumar et al. (1976) [211] studied the effect of foliar application of DMSO on rice plants (*Oryza sativa* L., variety Jaya) grown in a Zn-deficient soil. Application of ZnCl_2_ took place in the soil at the rates of 10 and 20 ppm, whilst foliar applications of DMSO took place at the rates of 0.001%, 0.01%, and 0.1%. Zn availability of the soil was increased by all DMSO treatments. Control plants showed very low dry matter. Applications of 0.001% and 0.01% of DMSO slightly increased dry weights of all plant parts at 45 days after transplanting. Grain yield was significantly increased by all DMSO treatments. Control plants showed very low chlorophyll contents. Chlorophyll content was stimulated by these doses of foliar application of DMSO, whilst the chlorophyll:carotenoid ratio was increased by all DMSO and Zn treatments. Control plants showed significantly lower activities of carbonic anhydrase and tryptophan synthetase. Carbonic anhydrase activity was significantly increased by the 0.001%, and 0.01% of the DMSO treatments, whilst tryptophan synthetase activity was stimulated only by the lowest dose of foliar (0.001%) applications [211].

(ii) DMSO and Fe. Foliar applications of various Fe-containing compounds—in most cases, inorganic Fe(II) and Fe(III) compounds—have been tested, usually with limited success. Criteria for assessing treatment success were regreening of yellow leaves, increases in Fe concentrations, as well as in chlorophyll and/or in leaves. This literature has been reviewed by Fernández and Ebert (2005) [11]. In an example provided by Shoenherr et al. (2005) [214], the penetration of FeSO_4_ at pH 3.9, 4.3, and 4.7 into maize leaves at 48–60% humidity has been investigated, where 0.5%, or 1% DMSO was included as an adjuvant and Tween 20 (0.02%) was used as the wetting agent. Very slow penetration was observed, and DMSO increased rates of the penetration of FeSO_4_. According to Singh and Kahn 2012 [215], the application of various water-soluble sources of Fe combined with DMSO markedly improved the Fe content, presenting rapid leaf greening and higher leaf chlorophyll contents of Fe chlorotic orange and grapefruit trees than without DMSO.

(iii) DMSO and phytohormones. The application of formulations with biostimulant action boost vegetable yields under typical and different biotic stress circumstances, whilst they are ecologically and user friendly. The production of highly stable emulsifiable concentrate (EC) formulations is a bottleneck of the uses of plant growth regulators, due to their hydrophobic character and huge molecular volumes. Ruidas et al. (2022) [216] studied phytohormone formulations of gibberellic acid with 0.25% EC, and brassinolide with 0.15% EC, using a variety of solvents, including DMSO and surfactants (calcium alkylbenzene sulfonate, or nonylphenol ethoxylate-13). Gibberellic acid boosted brinjal yields by 37.5%, while brassinolide raised onion yields by 33.9%. Brassinolide and gibberellin were both interdependent in action [216].

(iv) DMSO and agrochemicals. DMSO has been used as a systemic carrier of growth regulators, herbicides, and pesticides in plant tissues, by enhancing of the penetration of the applied substances into the tissues [217]. In a trial of foliar application of low concentrations of Cycocel [(2-chloroethyl) trimethyl ammonium chloride] to pea plants at the 5th node stage, the internode length, plant height, fresh plant weight, fresh and dry pea weight and total dry matter were increased. In combination with DMSO, Maurer et al. (1969) [218] found that pea plants could be sprayed safely with a 5% DMSO solution, whilst a 10% solution caused plant injury. In separate experiments at two locations with different climates and soils, DMSO was tested in field trials as a carrier for Cycocel. It was applied at 3 concentrations when pea plants were in the 5–6 node stage, with and without a 5% solution of DMSO. The effects of DMSO and Cycocel were additive. A surfactant [polyoxyethylene (20) sorbitan monolaurate] was included in all treatments [217].

A summary of the case studies on the foliar applications of S-containing non-metabolites discussed in this section is given in Table 2.

### 6.4. S-Containing Spray Adjuvants

Function of adjuvants. The foliar-applied compounds must penetrate through the epidermis. The term adjuvant or surfactant (meaning surface-active agent) characterizes any compound added into the spray solution, towards improving its performance and effectiveness for enhanced penetration. Such compounds reduce surface tension, alter the energy relationships at interfaces, and adjust themselves as interfaces as they contain both hydrophobic and hydrophilic group within their molecule [12,219].

Classification of S-containing adjuvants. The classification of the surfactants includes non-ionic, anionic, cationic, or molecules with ampholytic part, based on the presence and the nature of the electrical charge, or the absence of ionization on the hydrophilic portion of the molecule. The non-ionic surfactants are active depressants of surface-tension. Chemically they are inert due to lack of ionization and possess no charge groups in their heads. The hydrophobic group is associated with non-ionized hydrophilic groups as polymerized esters of polyether alcohols ethylene oxide, or polyhydric alcohols [220,221]. The heads of ionic surfactants carry net charge. The hydrophilic portion of an ionic surfactant can be either anionic (if the charge is negative) or cationic (if the charge is positive). Such surfactants are of limited relevance in agriculture since most nutrients are delivered as ionized compounds, which may interact and bind to the ionic surfactant molecules, thus altering their surface-active performance [1].

S-containing surfactants are anionic surfactants containing one or more functional groups. These groups become ionized in solution and generate the negatively charged organic ions that are responsible for lowering the surface tension. This class of surfactants includes alkyl-sulphates, alkyl-polyether sulfates, as well as paraffin-, olefin- and alkylbenzene-sulfonates and sulfate esters. The anionic S-containing surfactants may be sulfonates or sulfates [215]. The surfactants of the sulfonate class include docusates (dioctyl sodium sulfosuccinate), sulfonate fluorosurfactants (perfluorooctanesulfonate; perfluorobutanesulfonate), and alkyl benzene sulphonates. The surfactants of the sulfate class include alkyl sulfates (ammonium lauryl sulfate; sodium lauryl sulfate; sodium dodecyl sulfate), and alkyl ether sulfates (sodium laureth sulfate; sodium myreth sulfate). The sulfate ester groups (C-O-S) attaching the hydrophilic head to the surfactant are easily hydrolyzed to the corresponding alcohol and sulfate ion by dilute acids, whilst the stronger C-S bond of sulfonate groups is much more stable and will be broken only under extreme chemical conditions [221].

The surfactant that contains a head with two oppositely charged groups is the zwitterionic one. Such surfactants are compounds with a hydrophobic part, consisting of alkyl-substituted benzene, naphthalene or paraffinic chain ring and a hydrophilic group with a negatively charged carboxyl, sulfate, sulfonate, or phosphate group. This class of surfactants mainly include alcohols and/or fatty acids, which improve spreading, sticking and uptake of the sprayed materials due to lower surface tension. Cationic quaternary ammonium, arsonium, iodonium, phosphonium or sulfonium compounds have similar hydrophobic groups as in anionic surfactants. They can also link with positively charged hydrophilic group. The zwitterionic S-containing surfactants are either (i) sulfonates [CHAPS (3-[(3-Cholamidopropyl) dimethylammonio]-1-propanesulfonate)], or (ii) sultaines [cocamidopropyl hydroxysultaine] [1,215].

The ampholytic (or amphiphilic) surfactants present similar molecular arrangement as hydrophilic groups, with the capacity to become cationic in an acidic medium and anionic in a basic medium. The lack of ionization renders the amphiphilic surfactants inert, and proper for application in biological systems since they work as surface-tension depressants [222].

Lignosulfonates are bio-based surfactants. It is very interesting that LS did not require surfactants for their application, because LS are themselves bio-based surfactants. LS present amphiphilic nature. In foliar applications, they did not burn the leaves, and they present a stimulating effect on the vegetative growth of the plants [208]. The physicochemical behavior of lignosulfonates is affected by the monolignol composition, the distribution of the molecular weight, as well as the chemical modification. On the other hand, hydrophobicity is the indicator that relates composition and behavior of LS. The function and performance of LS are determined by their behavior in aqueous solution at surfaces and interfaces. In aqueous solution, several parameters can affect LS conformation, the colloidal state, and adsorption at surfaces or interfaces. These parameters include pH, temperature, concentration of other electrolytes, and the presence of organic solvents. These parameters may also affect the adsorption behavior of LS [207].

### 6.5. S-Containing Agrochemicals

Sulfur and plant health. Nutrient-induced resistance (NIR) is the contribution of the targeted nutrition in the protection of plants against pests and diseases (Bloem et al. 2005) [223]. Because of its complexity and the availability of effective pesticides, research in the field of NIR mechanisms has been poor. However, the practical significance of NIR is not of secondary importance. Mechanisms of disease control with nutrition have been discussed by Huber and Haneklaus (2007) [224]. During the 1990s, when clean air legislation came into force, the S deficiency developed into a widespread nutrient disorder. Since then, S was investigated with respect to various aspects, plant nutrition and plant health included. Understanding the mechanisms of NIR contributes to maintaining plant health, and to minimizing the input of pesticides in the conventional systems. S-containing metabolites that contribute to effective pathogen resistance are volatile S compounds, GSH, glucosinolates, phytoalexins, S-rich proteins, and the formation of elemental S. Bloem et al. (2005) [223] summarized the knowledge up to that point of time as regards the relationship of these metabolites to pathogenesis and the influence of S nutritional status on them. The concept of S-induced-resistance (SIR) is still developing, and the target of this research is to identifying metabolites, enzymes, and reactions, potentially activated by the S metabolism to combat pathogens. The S status of the crop affects various plant features, including the release of gaseous S compounds. These features influence the desirability of a crop for a variety of different crop-related organisms. Bloem et al. (2015) [225] summarized the progress of this knowledge that connects the effect of the S nutritional status of agricultural crops to their health status.

S-containing agrochemicals. On the other hand, S-containing xenobiotics play important roles in the control of weeds, insects, and plant diseases. Lamberth (2004) [226] has provided an overview of the significance of S-containing compounds in crop protection with chemicals and presented the main classes of organic agrochemical S-compounds. These xenobiotics present a broad range of modes of action. In some of them, the S atom plays an important role in the transformation of propesticides into active substances. On the other hand, several natural products bearing S atoms display distinctive pesticidal properties, with special role of S in propesticide action. The S-carrying pesticides are mainly in fungicides, herbicides, and insecticides [226].

The introduction of S into a biologically active molecule can dramatically modify its biological activity. Parameters that are affected include (i) blocking metabolic deactivation, (ii) binding to a target receptor or enzyme, and/or (iii) transporting the bioactive molecule from the point of application to the target site. Thus, the introduction of S atoms into an active ingredient is an important tool towards the modulation of the properties of novel chemical compounds with new modes of action, tailored for crop protection. Most S-containing pesticides undergo metabolic activation by reactions involving or initiated by oxidation. The introduction of a S-containing moiety may enhance the selectivity. Metabolic conversion of sulfides to sulfoxides and sulfones alters the reactivity, solubility, and ease of translocation of systemic pesticides. Recently, Devendar and Yang (2017) [227] highlighted the interest in active S-containing compounds, providing a comprehensive overview of selected leading S-containing pesticidal chemical families, including sulfonylureas, sulfonamides, sulfur-containing heterocyclics, thioureas, sulfides, sulfones, sulfoxides, and sulfoximines.

### 6.6. Potential Contribution of ABC-Transporters and Glutathione S-Transferases to the Transport of the S-Containing Compounds

It seems that ABC-transporters and glutathione S-transferases do have roles to the transport of the S-containing compounds, as both contribute to handling the penetration of xenobiotics within the various plant tissues. ABC transporters are involved in plant responses to different types of stress, as they function as transporters of xenobiotics, secondary metabolites, stress hormones or regulators of stress response genes [228]. ABC proteins were originally identified as transporters involved in the vacuolar deposition, the final detoxification process. Since then, it has been shown that the functions of this class of transporters extend far beyond detoxification. They are involved in diverse processes including surface lipid deposition, pathogen response, phytate accumulation in seeds, and transport of the phytohormones auxin and abscisic acid and plant hormones that regulate the overall development of plants, as well as transport of secondary metabolites, coating materials, and supportive materials. Therefore, ABC transporters contribute to organ growth, plant nutrition, plant development, response to abiotic stress, and the interaction of the plant with its environment [229,230]. Of these processes, we also highlight the contribution to the delivery of the required materials that construct the foliage surface.

On the other hand, glutathione S-transferases (GSTs; E.C.2.5.1.18) belong to a super-family of multifunctional proteins acting as detoxifying enzymes, among many other functions. GSTs are versatile enzymes catalyzing a wide range of reactions involving the conjugation of GSH to electrophilic compounds, that is to an electrophilic center contained within a small molecule acceptor, to form more soluble peptide derivatives. Hernandez Estevez and Rodríguez Hernández (2020) [231] have summarized cases showing that GSTs are involved in diverse aspects of biotic and abiotic stresses, as well as regulatory functions, and intracellular events such as, herbicide detoxification, signal transduction, plant protection against ozone damages, heavy metals, xenobiotics, transporting anthocyanins, hydroperoxide detoxification, auxin homeostasis, tyrosine metabolism, the regulation of apoptosis, primary, and secondary metabolisms, stress metabolism, herbicide detoxification and plant protection against ozone damages, and microbes’ infections. Among the functions of GSTs, is the removal of ROS, including superoxide radicals, hydroxyl radicals, alkoxy radicals, hydrogen peroxide and singlet oxygen. The above-mentioned list of actions supports the idea that perhaps GSTs are involved in the performance of foliar applications of S-containing compounds, thus rendering GSH as an important player and its foliar application of high importance.

## 7. Conclusions and Prospects

The S-containing compounds hold a distinguished place in the area of foliar applications due to the various mechanisms they contribute and affect within the plant. The array of the examined case studies clearly shows that the diversity in the way foliar application has been used makes it difficult to compare between the various experiments. The timing of application and the frequency of application are crucial factors. In the agricultural practice, the less the better; therefore, the success of the application is judged by the combination of (i) application once at the proper time, (ii) better yield, and (iii) better production, and (iv) affordable cost of the spray product, given the weathering at the time of application. A detailed understanding of the mechanisms and the regulation of transport is needed, especially in the interactions between the penetrating S-compounds and the existing compounds in the apoplastic space; also, on the transporters in action and their variety given the tissue. The combination of the various components of the spray solution and the dose are of critical importance in such applications. Foliar application of S-compounds in various combinations is an emerging area of agricultural usefulness. The S-containing compounds are not applied alone in spray solutions; and in the agricultural practice, the need for proper combination is of prime importance. Last but not least is the dose. The tables clearly show that the applied dose was based on preliminary experiments under the circumstances, but more work is needed on this aspect. The agronomic situation the product is designed to alleviate broadens the area of potential applications of the products enriched with S-containing compounds.

## Figures and Tables

**Table 2:** Summary of the case studies on the foliar applications of S-containing non-metabolites discussed in Section 6. TU: thiourea and DMSO: dimethyl sulfoxide.

Crop	Agricultural Situation	Compound	mg L^−1^ (or %)	Foliar Application	Adjuvant	Reference
canola	heat stress	TU	500	alone		[204]
wheat	bread	TU	500	alone		[205]
	nutritional quality		1000			
wheat	late sowing	TU	300	alone		[202]
			600			
wheat	B toxicity	TU	200	combined with NO		[199]
			400			
rice	Zn-deficient soil	DMSO	0.001%	alone		[211]
			0.01%			
			0.1%			
maize		DMSO	0.5%	combined with FeSO_4_	Tween-20	[214]
			1%			
brinjal		DMSO		combined with GA	Ca alkylbenzene	[216]
					sulfonate	
